# Long-term corneal recovery by simultaneous delivery of hPSC-derived corneal endothelial precursors and nicotinamide

**DOI:** 10.1172/JCI146658

**Published:** 2022-01-04

**Authors:** Zongyi Li, Haoyun Duan, Yanni Jia, Can Zhao, Wenjing Li, Xin Wang, Yajie Gong, Chunxiao Dong, Bochao Ma, Shengqian Dou, Bin Zhang, Dongfang Li, Yihai Cao, Lixin Xie, Qingjun Zhou, Weiyun Shi

**Affiliations:** 1 State Key Laboratory Cultivation Base, Shandong Provincial Key Laboratory of Ophthalmology, Shandong Eye Institute, Shandong First Medical University & Shandong Academy of Medical Sciences, Qingdao, China.; 2 Qingdao Eye Hospital of Shandong First Medical University, Qingdao, China.; 3 Eye Hospital of Shandong First Medical University, Jinan, China.; 4 Department of Microbiology, Tumor, and Cell Biology, Karolinska Institute, Stockholm, Sweden.

**Keywords:** Ophthalmology, Stem cells, Embryonic stem cells, Endothelial cells, Stem cell transplantation

## Abstract

Human pluripotent stem cells (hPSCs) hold great promise for the treatment of various human diseases. However, their therapeutic benefits and mechanisms for treating corneal endothelial dysfunction remain undefined. Here, we developed a therapeutic regimen consisting of the combination of hPSC-derived corneal endothelial precursors (CEPs) with nicotinamide (NAM) for effective treatment of corneal endothelial dysfunction. In rabbit and nonhuman primate models, intracameral injection of CEPs and NAM achieved long-term recovery of corneal clarity and thickness, similar with the therapeutic outcome of cultured human corneal endothelial cells (CECs). The transplanted human CEPs exhibited structural and functional integration with host resident CECs. However, the long-term recovery relied on the stimulation of endogenous endothelial regeneration in rabbits, but predominantly on the replacing function of transplanted cells during the 3-year follow-up in nonhuman primates, which resemble human corneal endothelium with limited regenerative capacity. Mechanistically, NAM ensured in vivo proper maturation of transplanted CEPs into functional CECs by preventing premature senescence and endothelial-mesenchymal transition within the TGF-β–enriched aqueous humor. Together, we provide compelling experimental evidence and mechanistic insights of simultaneous delivery of CEPs and NAM as a potential approach for treating corneal endothelial dysfunction.

## Graphical abstract

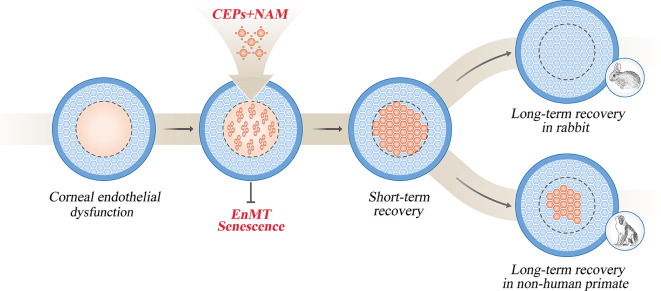

## Introduction

Corneal endothelium maintains corneal hydration and transparency through its barrier and pump function. When the endothelial cell density diminishes to fewer than 500 cells/mm^2^ due to dystrophy, trauma, or surgical intervention, corneal endothelial dysfunction will occur and lead to corneal edema, pain, and vision loss, known as bullous keratopathy (1, 2). Corneal transplantation represents the major therapeutic approach to treating bullous keratopathy, and it was limited by the global shortage of donor corneas (3, 4). Recently, intracameral injection of cultured corneal endothelial cells was reported to improve the visual acuity of patients for 5-year follow-up (5, 6). However, the expansion and identification of effector cells for transplantation remains the current challenge.

Stem cell–based therapy holds great promise for the treatment of corneal endothelial dysfunction (7). Previous reports have described the outcomes of corneal endothelial-like cells from somatic stem cells and reprogrammed fibroblasts in animal models (8–11), but the heterogenous efficiency restricts the development of standard methods for clinical application (12). Based on the pluripotent potential and self-renewal capacity, human pluripotent stem cells (hPSCs), including embryonic stem cells (ESCs) and induced pluripotent stem cells (iPSCs), have been used in multiple clinical trials for the treatment of cardiovascular, neurological, and ophthalmic diseases (13). Therefore, hPSCs may provide the promising cell sources to circumvent the severe shortage of donor corneas. Comparatively, autologous hiPSCs avoid the ethical and immune rejection issues associated with hESCs (14, 15).

For the induction of corneal endothelial cells from hPSCs, most studies focus on the in vitro morphology and immunostaining methods (16–25).One group investigated the in vivo therapeutic outcome of hESC-derived cells through the transplantation of tissue-engineered cornea (16, 17). More recently, the efficiency of corneal endothelial cells from hESCs and hiPSCs was further evaluated through intracameral injection (26, 27). Compared with rapid regeneration of corneal endothelium after injury in rabbit (28), nonhuman primate resembles human corneal endothelium with limited regenerative capacity (29). Therefore, nonhuman primates are more valuable to explore the long-term therapeutic benefits and mechanisms of hPSC-derived cells as preclinical models of treating corneal endothelial dysfunction.

In this study, we developed a therapeutic regimen consisting of the combination of hPSC-derived corneal endothelial precursors with nicotinamide for effective treatment of corneal endothelial dysfunction. When intracamerally injected in rabbit and nonhuman primate models, they achieved long-term recovery of corneal clarity and thickness, accompanied by the proper maturation and integration with host resident cells. The transplanted cells survived after 3-year follow-up in the nonhuman primate model. In addition, the long-term therapeutic mechanisms were compared between the rabbit and nonhuman primate models.

## Results

### Directed differentiation of hESCs into corneal endothelial precursors.

**Figure 1:**
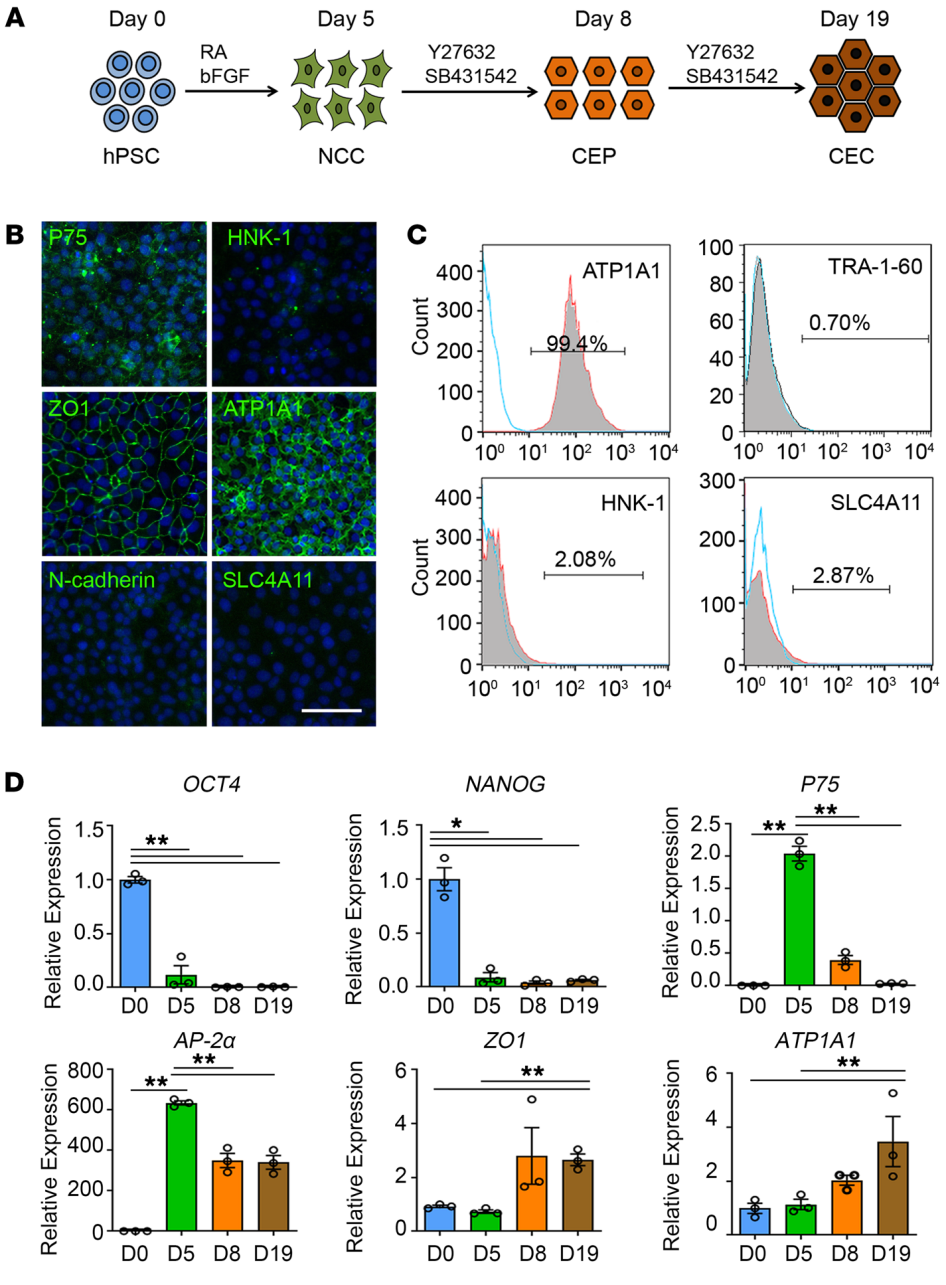
Induction and characterization of corneal endothelial precursors from hESCs. (**A**) Schematic representation of the method used for the generation of NCCs, CEPs, and CECs. For the NCC induction, hPSCs are incubated in the neural crest differentiation medium with 4 ng/mL bFGF and 1 μM RA for 5 days. For the CEP and CEC induction, the medium is changed into corneal endothelial differentiation medium with 10 μM Y27632 and 1 μM SB431542 for a subsequent 3 days and 14 days. (**B**) Representative immunofluorescence staining of the neural crest markers P75 and HNK-1, and corneal endothelial markers ZO-1, ATP1A1, N-cadherin, and SLC4A11 in CEPs. Nuclei were stained with DAPI. Scale bar: 50 μm. (**C**) Flow cytometry analysis of ATP1A1, TRA-1-60, HNK-1, and SLC4A11 in CEPs. The experiments were repeated 3 times. (**D**) qPCR analysis of the gene expression during the 3 stages of hESC differentiation. *n =* 3, **P < 0.01, *P < 0.05 by 1-way ANOVA with Tukey’s HSD test.

Through the recapitulation of corneal endothelial development, we developed a chemically defined method to induce the differentiation of neural crest cells (NCCs), corneal endothelial precursors (CEPs), and corneal endothelial-like cells (CECs) from hESC line H1 (Figure 1A). The hESCs stained positive for pluripotent markers OCT4 and NANOG (Supplemental Figure 1A; supplemental material available online with this article; https://doi.org/10.1172/JCI146658DS1). Following 5 days of neural crest induction, the cells exhibited cobblestone morphology with positive stained neural crest markers P75, HNK-1, AP-2α, and AP-2β (Supplemental Figure 1A). Flow cytometry analysis revealed that the purity of P75^+^/HNK-1^+^ cells reached 88.17% ± 0.93% (Supplemental Figure 1B). The NCCs exhibited multipotent capacity of generating peripheral neurons, corneal keratocytes, and mesenchymal stem cells (Supplemental Figure 2). The NCC-derived peripheral neurons stained positive for β-tubulin III and peripherin, and corneal keratocytes stained positive for keratocan, vimentin, and F-actin. The NCC-derived mesenchymal stem cells (MSCs) could differentiate into adipoblasts, osteoblasts, and chondroblasts, which were characterized by Oil Red O, Alizarin Red, and Alcian Blue staining, respectively (Supplemental Figure 2). After 14 days of corneal endothelial induction, the NCCs changed into hexagonal morphology with positive-stained corneal endothelial markers ZO1, ATP1A1, AQP1, and N-cadherin, but negative-stained for SLC4A11 (Supplemental Figure 1A).

During the inductive differentiation, we identified a middle-stage endothelial precursor after 3 days of corneal endothelial induction. These cells stained positive for neural crest markers P75 and AP-2β, and corneal endothelial markers ZO1, ATP1A1, and AQP1, but negative for HNK-1, N-cadherin, and SLC4A11 (Figure 1B and Supplemental Figure 1A). FACS analysis revealed that 99.28% ± 0.46% cells were ATP1A1 positive, while 0.70% ± 0.25% positive for TRA-1-60, 2.81% ± 1.75% positive for SSEA4, 2.90% ± 1.07% positive for HNK-1 and 2.03% ± 0.62% positive for SLC4A11 (Figure 1C and Supplemental Figure 3A). Moreover, the cells stained negative for OCT4 and NANOG and had no teratoma formation within 3 months of subcutaneous injection in NOD/SCID mice (Supplemental Figure 3, B–D). These results indicate that the cells have differentiated from neural crest stage and represent the fate-committed corneal endothelial precursors. In addition, we performed a time-course transcript analysis of the differentiated cells. Following rapid decline of pluripotent genes *OCT4* and *NANOG*, *P75* and *AP-2α* were upregulated after neural crest induction and subsequently downregulated after corneal endothelial differentiation, while *ZO-1* and *ATP1A1* were continuously elevated (Figure 1D). Immunostaining showed that the percentage of Ki67-positive cells gradually reduced with the duration of differentiation (Supplemental Figure 1, C and D). Collectively, these data suggest that the inductive differentiation recapitulates the progression of corneal endothelial development.

### Transient recovery with simple injection of hESC-derived cells and Y27632.

**Figure 2:**
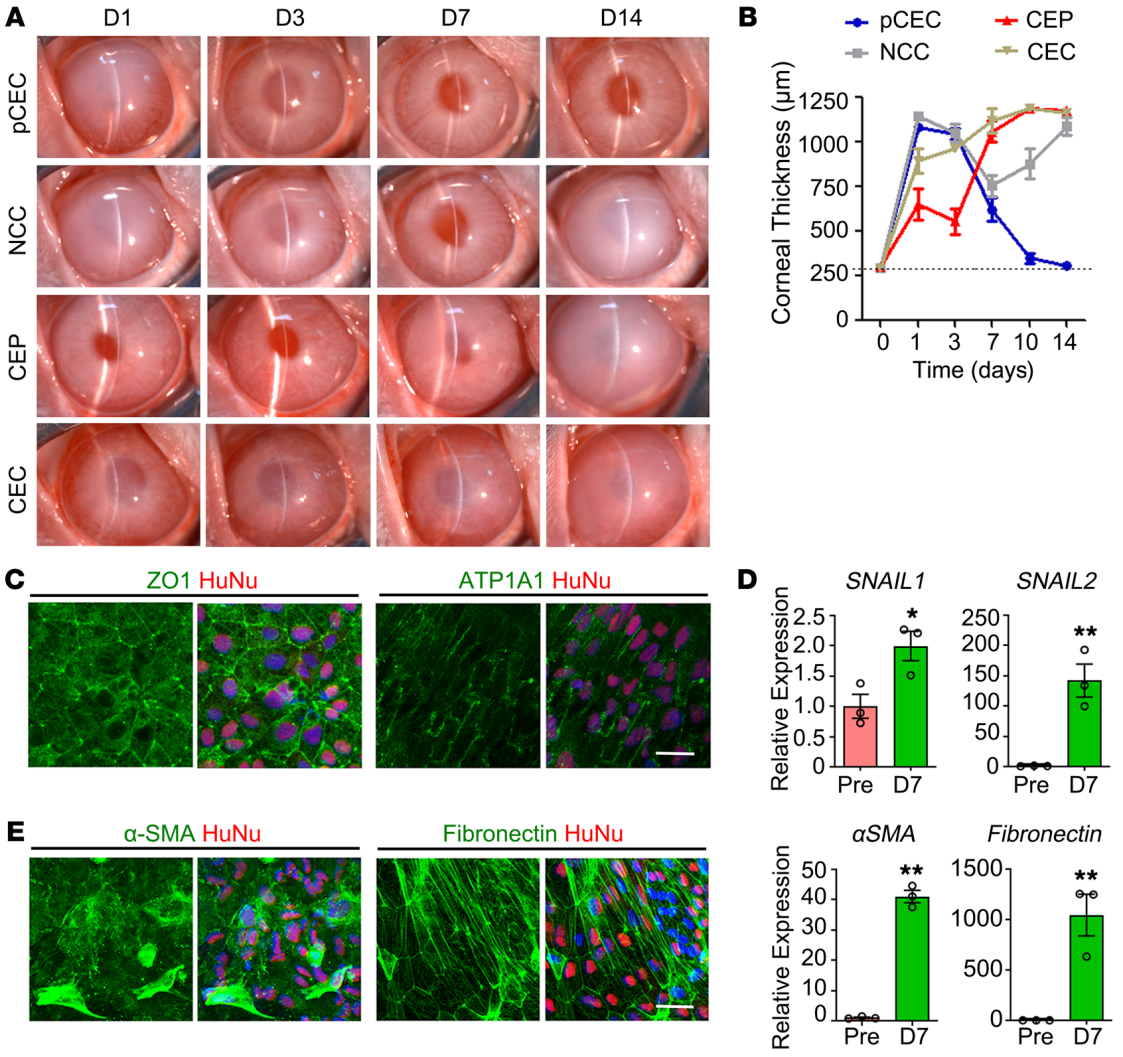
Comparisons of the hESC-derived cells for rabbit corneal recovery. (**A**) Human pCECs and hESC-derived NCCs, CEPs, and CECs were intracamerally injected in the rabbit corneal endothelial dysfunction model. Corneal transparency was assessed by slit-lamp microscopy after 1, 3, 7, and 14 days of transplantation. (**B**) Central corneal thicknesses were measured by pachymeter after 1, 3, 7, 10, and 14 days of transplantation. *n* = 3. The dashed line shows normal corneal thickness. (**C**) Immunostaining of ZO-1 and ATP1A1 in the transplanted CEPs 7 days after transplantation. The transplanted cells were stained by human specific antibody human nuclei (HuNu). Nuclei were stained with DAPI. Scale bar: 50 μm. (**D**) Expressions of EnMT markers in the pretransplanted (Pre) and transplanted CEPs 7 days after transplantation by qPCR. *n* = 3. *P < 0.05, **P < 0.01 by 2-tailed Student’s *t* test. (**E**) Immunostaining of α-SMA and fibronectin in the transplanted CEPs 7 days after transplantation. The transplanted cells were stained by HuNu. Nuclei were stained with DAPI. Scale bar: 50 μm.

To evaluate the therapeutic effects of differentiated cells, rabbit central corneal endothelium (9 mm diameter) was scraped from Descemet’s membrane as the model of corneal endothelial dysfunction (Supplemental Figure 4). A quantity of 8 × 10^5^ hESC-derived NCCs, CEPs, or CECs supplemented with 100 μM Y27632 was injected into the anterior chamber, with primary cultured human CECs (pCECs) as positive control. The rabbits injected with pCECs gradually resolved corneal edema with the recovery of normal clarity and thickness after 14 days. However, all rabbits injected with hESC-derived cells presented transient recovery and became re-edematous after 14 days, among which the CEPs exhibited better than NCCs and CECs (Figure 2, A and B). In addition, the rabbits injected with sham operation, medium, or Y27632 alone showed continuous edematous or slower improvement (Supplemental Figure 5).

To explore the cause of corneal re-edema, we performed whole-mounted staining with the human nuclei determinant HuNu and corneal endothelial markers ZO1 and ATP1A1. As shown in Figure 2C, the transplanted CEPs exhibited sporadic and irregular distribution of ZO1 and ATP1A1. As in previous reports of endothelial-mesenchymal transition (EnMT) in corneal endothelial diseases and cell transplantation (30–32), we examined the EnMT-related gene expression before and after transplantation. Compared with pretransplanted CEPs, the mRNA transcripts of *Snail1*, *Snail2*, *α-SMA*, and *fibronectin* significantly increased after 7 days of transplantation (Figure 2D), accompanied with fibroblastic morphology and strong staining of α-SMA and fibronectin (Figure 2E). These results suggest that the improper EnMT of transplanted CEPs may contribute to their functional impairment and cause corneal re-edema after initial improvement.

### Stable recovery with simultaneous delivery of hESC-derived CEPs and nicotinamide.

**Figure 3:**
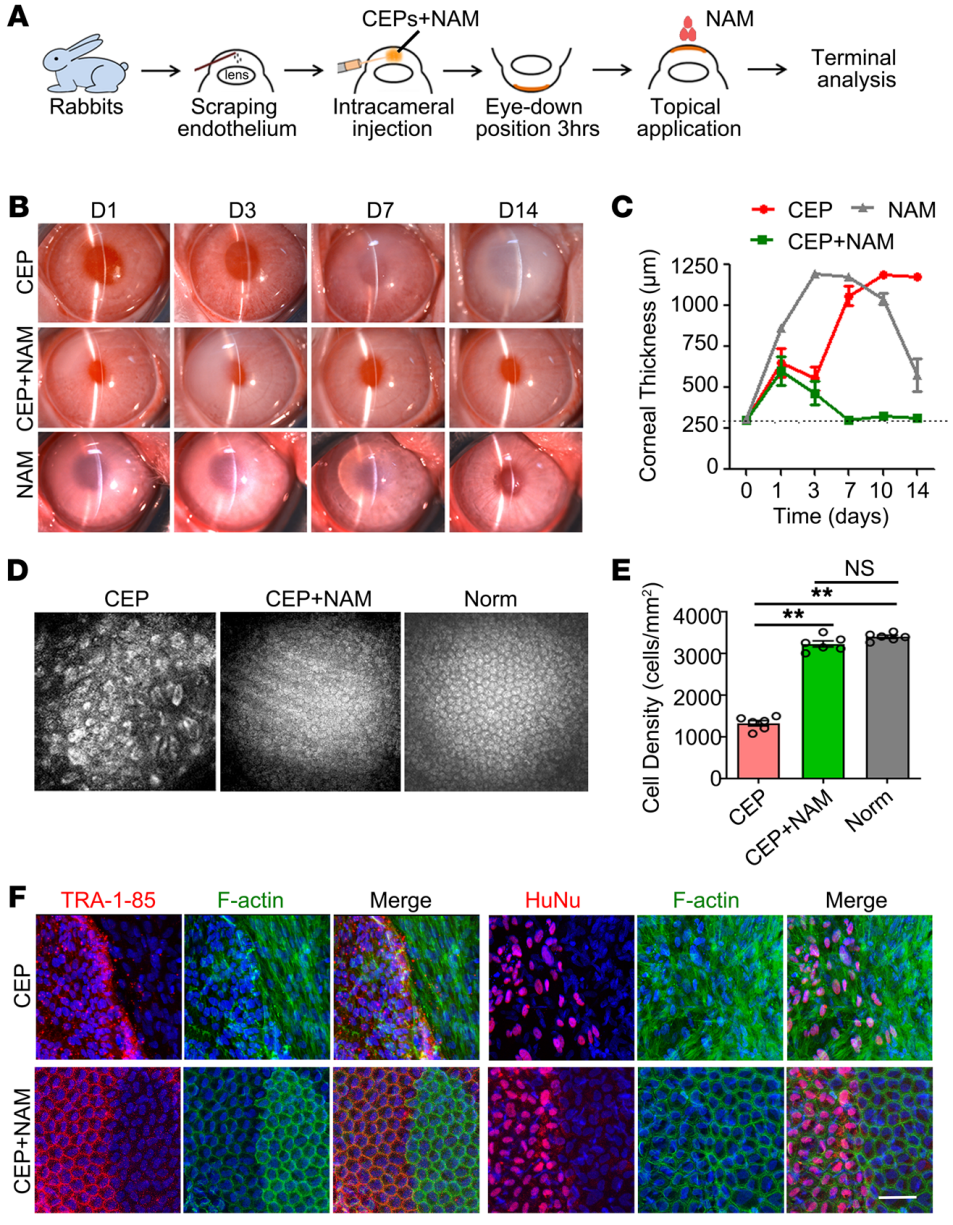
Efficacy of hESC-derived CEP injection combined with NAM treatment for rabbit corneal recovery. (**A**) Schema of the intracameral injection of hESC-derived CEPs and NAM treatment in the rabbit model of corneal endothelial dysfunction. (**B**) Corneal transparency was assessed by slit-lamp microscopy in rabbits with simple cell injection (CEP), cell injection and NAM treatment (CEP+NAM), and NAM alone treatment (NAM) 1, 3, 7, and 14 days after transplantation. (**C**) Central corneal thicknesses were measured by pachymeter 1, 3, 7, 10, and 14 days after transplantation. *n* = 6. The dashed line shows normal corneal thickness. (**D**) Regenerated corneal endothelium was evaluated by corneal confocal microscopy 7 days after transplantation, with the normal rabbit corneal endothelium (Norm) as control. (**E**) Statistical analysis of corneal endothelial density 7 days after transplantation. *n* = 6 **P <0.01 by 1-way ANOVA with Tukey’s HSD test. (**F**) Double staining of F-actin and human cell surface determinant TRA-1-85 and HuNu 14 days after transplantation. Nuclei were stained with DAPI. Scale bar: 50 μm.

Given the role of nicotinamide (NAM) in stem cell differentiation, survival, and EMT inhibition (33–35), we explored whether NAM can improve the therapeutic effect of CEP injection. Therefore, the rabbits were injected with 8 × 10^5^ cells containing 50 mM NAM and 100 μM Y27632, and 500 mM NAM and 10 mM Y27632 eye drops were applied 4 times per day (Figure 3A), with CEP injection, NAM alone without Y27632, and CEP plus NAM without Y27632 as controls. Consistently, all control rabbits showed corneal edematous or slower improvement (Figure 3, B and C and Supplemental Figure 5). However, when combined with NAM, CEP injection rapidly improved corneal clarity and restored normal thickness at day 7 (Figure 3, B and C). Confocal microscopy observation showed that the rabbit corneas possessed intact endothelium with relative regular morphology and favorable endothelial cell density (Figure 3, D and E), but the transplanted cells didn’t achieve uniform alignment as host resident cells (Supplemental Figure 6). The recovery was continuously maintained during the follow-up to 8 weeks (Supplemental Figure 7A). No significant changes of intraocular pressure were observed (Supplemental Figure 8). The human specific *Actin* was detected only in the corneal endothelium and trabecular tissue within the first 4 weeks (Supplemental Figure 9).

To evaluate the survival and integration of transplanted cells, we performed double immunofluorescence staining with the human nuclei determinant HuNu and cell-surface determinant TRA-1-85, combined with phalloidin to label cytoskeleton F-actin. The transplanted cells covered the scraped area within 2 weeks, decreased after 4 weeks, and were completely lost after 8 weeks. The proliferating cell nuclear antigen–positive (PCNA-positive) cells were detected among the host resident corneal endothelium (Supplemental Figure 7). More specifically, the transplanted CEPs with NAM treatment formed a regularly aligned monolayer and integrated with host cells at day 14, although partial cells exhibited weaker F-actin staining than host endothelium (Figure 3F and Supplemental Figure 10). By contrast, in rabbit corneas without NAM treatment, both transplanted cells and neighboring host cells displayed the fibroblastic morphology (Figure 3F), suggesting the inhibitory effect of NAM on EnMT as our previous description (35). When NAM was withdrawn after CEP injection, corneal re-edema appeared in 40% of the rabbits, indicating continuous NAM treatment is essential for the maintenance of corneal recovery (Supplemental Figure 11). Collectively, these results suggest that short-term recovery is achieved through the contribution of transplanted CEPs and NAM, but that long-term recovery relies on the stimulation of resident endothelial regeneration with the loss of transplanted cells.

### NAM orchestrates in vivo functional maturation of hESC-derived CEPs.

**Figure 4:**
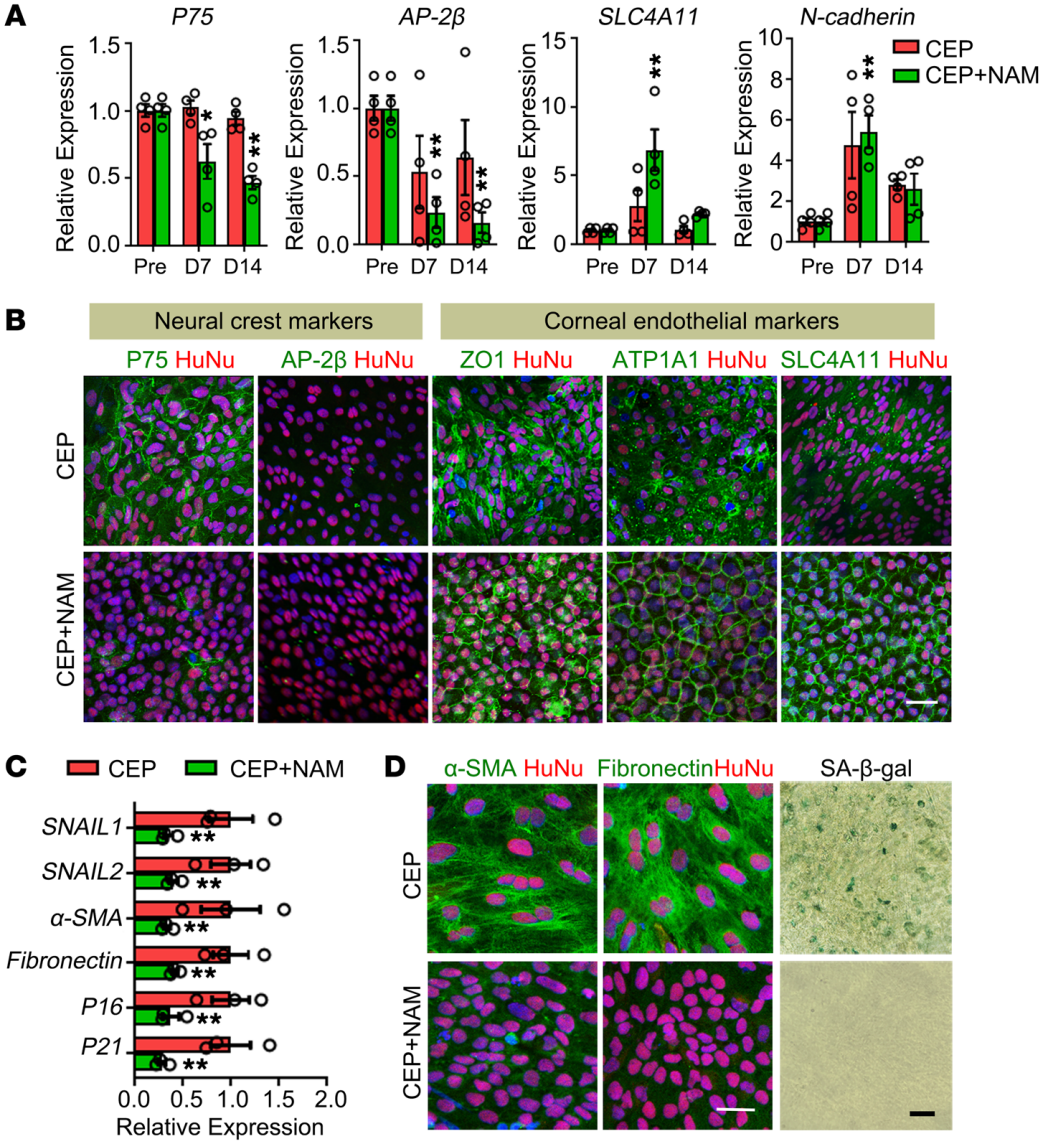
Effects of NAM treatment on transplanted corneal endothelial precursors. (**A**) Expressions of neural crest and corneal endothelial genes in the pretransplanted (Pre) and transplanted cells at day 7 and day 14 after transplantation by qPCR using human-specific primers. *n* = 4. *P < 0.05, **P < 0.01 versus the pretransplanted CEPs. (**B**) Double staining of neural crest markers P75, AP-2β, and corneal endothelial markers ZO-1, ATP1A1, and SLC4A11 at day 14 after transplantation. Cells from human origin were stained with HuNu. Nuclei were stained with DAPI. Scale bar: 50 μm. (**C**) Expressions of EnMT and senescence-associated genes at day 7 after transplantation by qPCR. *n* = 3, **P < 0.01. (**D**) Double staining of α-SMA and fibronectin, and SA-β-gal staining at day 7 after transplantation. The transplanted cells of human origin were stained by HuNu. Nuclei were stained with DAPI. Scale bar: 50 μm. One-way ANOVA with Tukey’s HSD test (**A**) and 2-tailed Student’s *t* test (**C**).

To explore the potential mechanism of NAM treatment, we collected the corneal endothelium within the scraped area and compared the expression patterns of transplanted cells using human-specific primers. The expressions of neural crest genes decreased gradually, while corneal endothelial genes increased after transplantation (Figure 4A and Supplemental Figure 12A). The transplanted cells stained positive for neural crest marker P75 and discontinuous corneal endothelial markers ZO-1, ATP1A1, and SLC4A11 at day 7 (Supplemental Figure 12B). After 14 days of transplantation with NAM treatment, they stained negative for P75 and AP-2β, and displayed an intact positive staining pattern for corneal endothelial markers, while the cells without NAM still exhibited sparse and irregular staining (Figure 4B). Furthermore, NAM treatment reduced the mRNA transcripts of EnMT- and senescence-associated genes (Figure 4C). The transplanted cells exhibited negative staining of α-SMA and fibronectin as well as the reduced senescent cells (Figure 4D).

Previous studies have reported the involvement of TGF-β signaling in the senescence and EnMT of corneal endothelial cells (36–37). Therefore, we collected the rabbit aqueous humor and found increased TGF-β1 levels after endothelial scraping (Supplemental Figure 13). The inhibition of NAM on TGF-β1–induced EnMT and cellular senescence was confirmed by the in vitro cultured hESC-derived CEPs (Supplemental Figure 14A). RNA microarray analysis showed significant changes with NAM treatment (Supplemental Figure 14B). The core member in TGF-β signaling pathway, *TGFB2*, 2 classical EnMT related genes, *ACTA2* (α-SMA) and *FN1* (fibronectin), and 2 cellular senescence–associated genes, including *P16* and *P21*, declined with NAM treatment, while the corneal endothelial gene *COL8A2* and several anti-EnMT genes were elevated (38–40). KEGG analysis revealed that differentially expressed genes were involved the TGF-β signaling pathway, cellular senescence, and focal adhesion (Supplemental Figure 14C). Further analysis and qPCR validation revealed that NAM repressed TGF-β and cellular senescence–related gene sets, and induced the upregulation of focal adhesion–associated genes (Supplemental Figure 14, D and E). Overall, these results suggest that NAM orchestrates in vivo functional maturation of hESC-derived CEPs through multiple mechanisms, similar to previous descriptions (33, 34, 41).

To further corroborate the therapeutic strategy, we repeated the inductive method and intracameral injection with 2 human iPSC lines DYR0100 and U2. The transplantation of hiPSC-derived CEPs and NAM resolved corneal edema with the recovery of corneal clarity and thickness in rabbits. The transplanted cells displayed regularly arranged staining of corneal endothelial markers (Supplemental Figure 15).

### Long-term outcomes of hESC-derived CEP injection and NAM in primate models.

**Figure 5:**
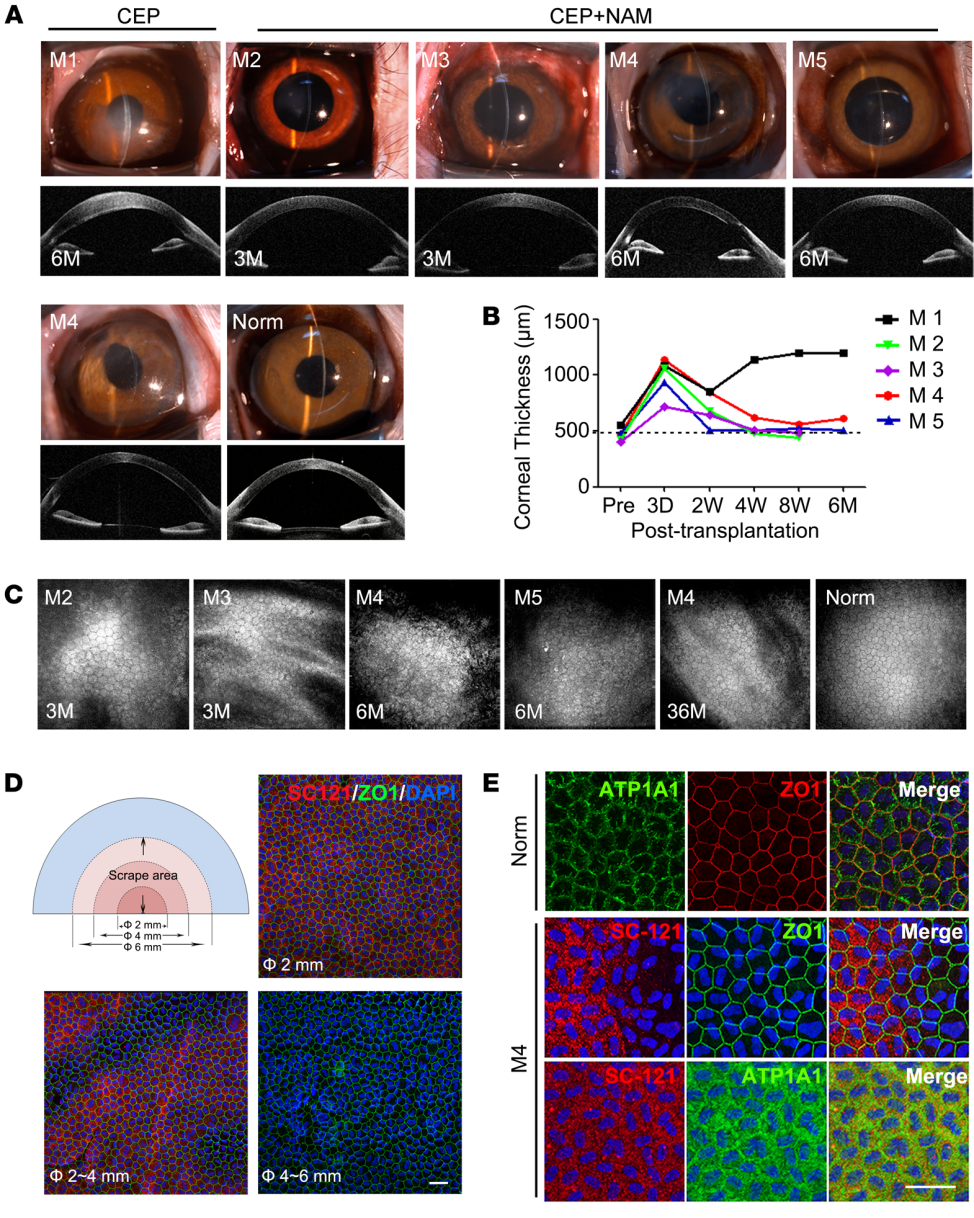
Therapeutic effect of hESC-derived CEPs with NAM treatment for corneal recovery in monkeys. (**A**) Corneal transparency and anterior segment OCT images of monkeys with CEP injection with (M2–M5) or without NAM treatment (M1) 3 months, 6 months, and 36 months after operation. Normal monkey (Norm) was used as the control. (**B**) Corneal thicknesses were measured by pachymeter at preoperation (Pre), 3 days, 2 weeks, 4 weeks, 8 weeks, and 6 months after transplantation. The dashed line shows normal corneal thickness. (**C**) Corneal confocal microscopy observation 3 months, 6 months, and 36 months after transplantation. No images of M1 without NAM treatment were obtained due to corneal edema. (**D**) Schematic representation of the scrape area in the half cornea of M4 with CEP and NAM treatment 36 months after operation. The 6 mm diameter scraped region was divided into 3 areas: Φ ≤ 2 mm, 2 mm ≤ Φ ≤ 4 mm, and 4 mm ≤ Φ ≤ 6 mm. Immunostaining of human cell–specific marker SC-121 and corneal endothelial marker ZO-1 in the different areas of the monkey corneal endothelium is shown. Nuclei were stained with DAPI. Scale bar: 50 μm. (**E**) Double staining of human cell–specific marker SC-121 and corneal endothelial markers ZO-1 and ATP1A1 in corneal endothelium of M4 36 months after transplantation. Nuclei were stained with DAPI. Scale bar: 50 μm.

Considering the limitation of the rabbit model (28), a nonhuman primate model was used to further elucidate the therapeutic benefits of hESC-derived CEP injection and NAM treatment. Central corneal endothelium (6 mm diameter) of 5 cynomolgus monkeys was scraped to generate the preclinical model. One monkey (M1) was injected with 6 × 10^5^ CEPs, 4 monkeys (M2–M5) were injected with CEPs and NAM treatment. During the follow-up of 3 and 6 months, M1 showed persistent corneal edema, while M2–M5 resolved corneal edema with recovered corneal clarity and thickness within 2–8 weeks, although the initial surgical operation caused iris distortion in M4 (Figure 5, A and B). Confocal microscopy confirmed that the corneal endothelium (M2 and M3 for 3 months, M4 and M5 for 6 months) displayed a relative regular morphology with the density of 2362 ± 108.25 cell/mm^2^ (M2), 2887 ± 54.49 cell/mm^2^ (M3), 2600 ± 154.11 cell/mm^2^ (M4), and 2050 ± 127.48 cell/mm^2^ (M5) (Figure 5C). B-mode ultrasound, fundus photography, and intraocular pressure confirmed no abnormal pathological changes (Supplemental Figure 16).

To verify the long-term outcome, we followed the M4 up to 36 months. Transplanted cells were not detected in the iris, trabecular, and internal organs as confirmed by PCR (Supplemental Figure 17A). The recovered corneal clarity and thickness were maintained with more regular endothelial morphology and cell density of 2431 ± 152.45 cell/mm^2^, which was greater than 70% of the normal eye (Figure 5, A–C). To evaluate the survival of transplanted cells, we screened several human antibodies, including HuNu, TRA-1-85, MTCO2, and SC101 (Supplemental Figure 18), and identified the human-specific antibody SC-121 to discriminate the transplanted cells from monkey resident cells. As shown in Figure 5D, the transplanted cells were detected within the complete central area of 2 mm diameter and partial middle area of 2–4 mm diameter. However, they were not detected in the peripheral area of 4–6 mm diameter, which was covered with only monkey cells. The PCNA-positive cells were also detected in the peripheral area after CEP transplantation and NAM treatment (Supplemental Figure 17B). Moreover, transplanted cells exhibited regular hexagonal morphology and corneal endothelial marker expression pattern, similar to the normal corneal endothelium (Figure 5E). These results suggest that the strategy of CEP transplantation and NAM treatment achieves the long-term recovery of corneal clarity and thickness in nonhuman primates. Although it stimulates the partial regeneration of resident monkey cells, major therapeutic effects predominantly rely on the replacing function of transplanted human cells.

## Discussion

hPSCs provide a promising alternative cell source for treating corneal endothelial dysfunction. Although several studies have reported that corneal endothelial differentiation is induced from hPSCs, the long-term therapeutic benefits and mechanisms remain unclear, especially in a nonhuman primate model with similar limited regenerative capacity to human corneal endothelium. In the present study, we generated the fate-committed corneal endothelial precursors from 1 hESC line and 2 hiPSC lines. When combined with NAM treatment, intracameral injection of hPSC-derived CEPs achieved long-term effective treatment of corneal endothelial dysfunction in rabbit and nonhuman primate models. Mechanistically, NAM ensured in vivo proper maturation of transplanted CEPs into functional CECs by preventing their EnMT and premature senescence.

The corneal endothelium is derived from cranial neural crest cells, which migrate and direct into corneal endothelial fate under the local microenvironment (42, 43). Similar to previous reports (18, 20, 21), we developed a 2-step method to induce the differentiation of neural crest cells and corneal endothelial-like cells from hESCs. However, when injected in rabbit model, the corneal endothelial-like cells showed almost no improvement, while the neural crest cells achieved transient corneal recovery. Therefore, we identified the middle-stage corneal endothelial precursors. Benefiting from the high purity of neural crest cells differentiated from hESCs, the CEP population was relative homogenous according to immunostaining and FACS analysis. Moreover, CEPs express neural crest markers P75 and AP-2β and corneal endothelial markers ZO-1, ATP1A1, and AQP1. Meanwhile, they have lost HNK-1 expression and are negative for mature corneal endothelial markers SLC4A11 and N-cadherin. The above evidence suggests that the CEP population represents putative fate-committed corneal endothelial precursors from neural crest cells.

The ROCK inhibitor Y27632 has been used in the intracameral injection of human CECs to promote their attachment and survival (44). Accordingly, we injected hESC-derived CEPs with Y27632 in rabbit models and found better improvement than NCCs and CECs within 3 days. However, the corneas became re-edematous because of TGF-β–induced EnMT of the transplanted cells. These data indicate that Y27632 alone is insufficient to maintain the in vivo long-term outcome of hESC-derived CEPs. Therefore, we selected NAM as another adjunctive supplement for transplantation. In line with a previous report (35), NAM alone promoted rabbit corneal endothelial wound healing, but the recovery of corneal clarity and thickness was slower and incomplete within 14 days. Comparatively, the CEP transplantation and NAM treatment achieved a rapid and complete recovery within 7 days, accompanied by a structural and functional integration with host cells. It should be mentioned that partial cells showed weaker F-actin than host resident cells, indicating that they were still immature after 14 days of transplantation in rabbits, even when normal corneal clarity and thickness restored completely.

Previous studies have described that stem/progenitor cell transplantation may promote the regeneration of host resident cells (45, 46). To demonstrate the therapeutic mechanisms of CEP transplantation and NAM treatment, we compared the short-term and long-term fate decision of transplanted cells in rabbits and nonhuman primates. In rabbits with rapid endothelial regenerative capacity, the strategy of CEP transplantation and NAM treatment achieved persisted corneal recovery from 7 days to 8 weeks. However, the transplanted cells were completely lost at 8 weeks, accompanied the presence of dividing cells in resident corneal endothelium. This evidence supports the theory that short-term corneal recovery in rabbits is achieved through the replacing function of transplanted cells, while long-term recovery relies on the stimulated regeneration of resident cells. In nonhuman primate models with limited endothelial regenerative capacity, the strategy of CEP transplantation and NAM treatment also achieved persisted corneal recovery from 8 weeks to 36 months. When checking the survival of transplanted cells at 36 months, we found the peripheral scraped area was covered with only monkey cells, the middle area was mixed with both monkey and transplanted human cells, and the central area was covered with only transplanted human cells. These findings support that the transplanted cells actually stimulate the limited regeneration of monkey resident cells, similar to a previous description of descemetorhexis without endothelial keratoplasty in human (47–49). However, the long-term corneal recovery predominantly relies on the replacing function of transplanted human cells, which was different from the dependence of stimulated endothelial regeneration in rabbits.

It should be mentioned that there are still some limitations for the clinical translation of CEP transplantation and NAM treatment, such as complete transparency recovery, stromal haze, and irregular endothelial morphology. The initial intraocular inflammation represents a major problem for the success of corneal recovery in nonhuman primate models. Although the moderate-to-severe inflammatory response was found in 1 monkey, once controlled rapidly, no further inflammation and rejection occurred during the 3-year follow-up.

In summary, this study described a therapeutic regimen consisting of the combination of hPSC-derived CEPs and nicotinamide for the treatment of corneal endothelial dysfunction. Nicotinamide promoted the proper maturation of transplanted CEPs into functional CECs by preventing EnMT and premature senescence within the TGF-β–enriched aqueous humor. Long-term corneal recovery relied on the replacing function of transplanted cells in nonhuman primate model.

## Methods

### Animals.

One-year-old male New Zealand white rabbits (Kangda Biotechnology) and 3- to 5-year-old female cynomolgus monkeys (Guidong Quadrumana Development & Laboratory Co.) were used to establish the model of the corneal endothelial dysfunction for transplantation experiment. Six- to eight-week-old male NOD/SCID mice (Vital River Laboratory Animal Technology Co.) were used for the teratoma formation experiment.

### Cell lines.

The human ES cell line H1 was provided by Zhengqin Yin. The human iPS cell line DYR0100 and U2 were provided by the Stem Cell Bank, Chinese Academy of Sciences and Beijing Cellapy Biotechnology Company. The hESCs and hiPSCs were cultured in serum-free medium mTeSR1 (STEMCELL Technology) in plates coated with growth factor–reduced Matrigel (BD Biosciences). The hESCs and hiPSCs were manually passaged once every 4 to 5 days with Accutase (Sigma). All cells were incubated at 37°C in a humidified atmosphere containing 5% CO_2_. The medium was changed every day.

### Directed differentiation of NCCs, CEPs, and CECs from hPSCs.

The hPSCs (2.5 × 10^5^ cells) were seeded on 1% Matrigel-coated dishes and grew into 30% confluence after 4 days’ culture, then cultured in neural crest differentiation medium (NDM) consisting of DMEM/F12 (Gibco), 20% knockout serum replacement (KSR, Gibco), L-GlutaMAX (2 mM, Gibco), MEM nonessential amino acids (0.1 mM, Gibco), β-mercaptoethanol (0.1 mM, Gibco), basic fibroblast growth factor (4 ng/mL, bFGF, R&D Systems), and 1 μM retinoic acid (RA, Sigma). After 5 days of culture, the differentiated cells grew into approximately 80% confluence and the medium was replaced with corneal endothelial differentiation medium (CDM) consisting of DMEM/F12 (Gibco), 0.2% BSA, bFGF (8 ng/mL), PDGF-BB (10 μg/mL, R&D Systems), DKK-2 (10 μg/ml, R&D Systems), insulin-transferring-selenium (Gibco), 2 mM L-GlutaMAX (Gibco), NEAA (0.1 mM), ascorbic acid (50 μg/mL, Sigma), Heregulin β-1 (10 ng/mL, Peprotech), IGF-1 (200 ng/mL, Peprotech), 50 × B27 (Gibco), β-mercaptoethanol (0.01 mM, Sigma), Y27632 (10 μM, Sigma), and SB431542 (1 μM, Millipore). The NCCs were induced into CEPs in the following 3 days, and toward CECs within 14 days. All cells were incubated at 37°C in a humidified atmosphere containing 5% CO_2_ and the medium was changed every day.

### Peripheral neuron, mesenchymal cell, and corneal keratocyte differentiation.

For peripheral neuron differentiation, NCCs were cultured in neuron differentiation media containing DMEM/F12, N2 supplement (Gibco), BDNF (10 ng/mL, R&D Systems), GDNF (10 ng/mL, R&D Systems), NGF (10 ng/mL, Peprotech), neurotrophin-3 (10 ng/mL, Peprotech), sodium l-ascorbic acid salt (200 μM) and dbcAMP (0.5 mM, Sigma) for 12–14 days. The medium was changed every 2 days. For mesenchymal differentiation, hiPSC-NCCs were cultured in media containing DMEM/F12, 10% FBS (Gibco), 1% penicillin-streptomycin (Corning), 1% l-alanyl-l-glutamine (Gibco), and 2-mercaptoethanol (0.1 mM, Sigma). The differentiated cells were passaged every 4 to 5 days and the media were changed every 2 days. Adipoblast, osteoblast, and chondroblast differentiation were performed according to the manufacturer’s directions using MesenCult Osteogenic Differentiation Kit (catalog 05465), MesenCult Adipogenic Differentiation Kit (catalog 05412), and MesenCult-ACF Chondrogenic Differentiation Kit (catalog 05455) (STEMCELL Technologies), respectively. For corneal keratocyte differentiation, NCCs were cultured in matrigel-coated plates and keratocyte differentiation media containing DMEM/F12, FGF2 (10 ng/mL), ascorbic acid-2-phosphate (1 mM, Sigma), 1% ITS, and 1% NEAA.

### Teratoma formation.

A total of 5 × 10^6^ hPSC-derived CEPs or hESCs were mixed with Matrigel (Corning), and subcutaneously injected into one flank of the NOD/SCID mice. Tumors were isolated at 3 months after injection, fixed in 4% formaldehyde solution, and embedded in paraffin. Sectioned tumors were stained with hematoxylin and eosin.

### Flow cytometry.

The differentiating cells derived from hPSCs were dissociated using EDTA-trypsin (Sigma) and stained with antibodies against P75 (Biolegend) and HNK-1 (Biolegend) as neural crest markers, antibodies against ATP1A1 (Biolegend) and SLC4A11 (Bioss) as corneal endothelial markers, or antibodies against the pluripotent markers TRA-1-60 (Biolegend) and SSEA4 (Biolegend) for 30 to 60 minutes on ice. Premier Data Acquisition and Analysis software (BD Biosciences) was used for analysis. Data were analyzed using FlowJo software (TreeStar).

### Immunostaining.

At various time points after differentiation or cell transplantation, the differentiated hPSCs and the full-thickness flat mounts of the corneas obtained from the animal models were fixed in 4% PFA. The samples were blocked with 5% normal serum for 30 minutes at room temperature and treated with the primary antibodies for overnight at 4°C (Table 1), and subsequently with Alexa Fluor 488– and Alexa Fluor 594–conjugated secondary antibody (Invitrogen) for 1 hour at 37 °C. Nuclei were stained with DAPI (Beyotime Biotechnology) before fluorescence microscopy imaging (Nikon, Japan and Zeiss LSM880, Germany).

### Quantitative reverse transcription PCR.

Total RNA was extracted from the differentiated and transplanted cells and the different tissues by using MiniBEST Universal RNA Extraction Kit (TaKaRa) at different time points after differentiation and cell transplantation. The transplanted cells in the scraped area of corneal endothelium were indicated by the 7 mm ring drill, and subsequently collected for qPCR detection. cDNAs were synthesized using a Primescript RT Reagent Kit (TaKaRa) according to the manufacturer’s protocol. Real-time PCR was carried out using SYBR Green reagents on an Applied Biosystems 7500 Real Time PCR System (Applied Biosystems). The cycling conditions were 10 seconds at 95°C followed by 40 two-step cycles (15 seconds at 95°C and 1 minute at 60°C). The quantified data were analyzed using Sequence Detection System software (Applied Biosystems) with GAPDH or actin as internal control (Table 2).

### Senescence-associated β-galactosidase assay.

SA-β-gal staining was performed according to the manufacturer’s protocols (Cell Signaling Technology). The corneas were isolated from rabbits 7 days after transplantation. The full-thickness corneal flat mounts and the treated cells were fixed for 15 minutes in 1× fixative solution followed by PBS washing and then incubated overnight with a staining solution in a dry incubator at 37°C in 4% PFA. The cells were viewed under bright field microscopy (Echo Laboratories).

### Cell transplantation and in vivo examinations.

The rabbits were anesthetized with intramuscular ketamine hydrochloride (40 mg/kg, Gutian Pharmaceutical Co.) and pelltobarbitalum natricum (50 mg/kg, Sinopharm Chemical Reagent Co.). The cynomolgus monkeys were anesthetized with intramuscular ketamine hydrochloride (20 mg/kg) and chlorpromazine hydrochloride (20 mg/kg, SPH No. 1 Biochemical & Pharmaceutical Co.). The 9 mm diameter central corneal endothelium of one eye of each rabbit was gently scraped from the Descemet’s membrane by an experienced corneal surgeon with a 20-gauge soft tapered silicone needle (Inami), and the 6-mm diameter central corneal endothelium was scraped in the nonhuman primate monkey model. Sodium hyaluronate was intracamerally injected to maintain the anterior chamber during debridement. The sodium hyaluronate and cell debris were rinsed by physiological saline after debridement procedure, and then 250 μL heparin sodium injection (625 U/mL, Qianhong Bio-pharma) was injected into anterior chamber to reduce the exudation. The lenses of all animals were preserved.

The cells were dissociated with Accutase for 15 minutes and then gently triturated into cell suspension. Subsequently, 8 × 10^5^ cells were suspended in DMEM (250 μL, Corning) supplemented with Y27632 (100 μM, Sigma) with or without NAM (50 mM, Sigma) to be injected into the rabbit models. Similarly, 6 × 10^5^ cells were suspended for injection into the nonhuman primate monkey models. The animals were kept in the eye-down position for 3 hours to allow the rapid attachment of cells. The treated eyes of the rabbits received topical administration of Y27632 (10 mM) diluted in PBS 4 times daily as previously described (50). NAM (500 mM) diluted in PBS was applied topically to the eyes of the rabbits injected with CEPs and NAM for 2 to 8 weeks. The rabbits received tobramycin and dexamethasone eye drops and pralofen eye drops 4 times daily. The cynomolgus monkeys received daily administrations of cyclosporine A (8 mg/kg, orally) 2 days prior to cell implantation and every 2 days for 4 weeks after transplantation. After transplantation, the monkeys received dexamethasone phosphate tablet (5 mg, orally) daily, and the treated eyes of the monkeys received topical administrations of Y27632 (10 mM), tobramycin, and dexamethasone eye drops and pralofen eye drops 4 times daily for 4 weeks. NAM (250 mM) was applied topically to the eyes of the monkeys injected with CEPs and NAM every 2 days for 4 weeks.

Corneal transparency and endothelial cell morphology were evaluated by slit-lamp microscopy (Topcon) and corneal confocal microscopy (Heidelberg Engineering), respectively. Corneal endothelial density was analyzed by corneal endothelial microscopy (Konan Medical). Corneal thickness was measured by a hand-held pachymeter (Tomey), and the highest detection level was 1200 μm. The intraocular pressure was examined with a tonometer (Tono-Pen AVIA). The anatomical structure of the cornea was verified by anterior segment optical coherence tomography (OCT) (Tomey).

### RNA microarray analysis.

Total RNA was extracted from CEPs treated with or without NAM (5 mM) in the presence of TGFβ1 (30 ng/mL, R&D Systems) after a 3-day culture. Total RNAs were quantified by the NanoDrop ND-2000 (Thermo Fisher Scientific) and the RNA integrity was assessed using Agilent Bioanalyzer 2100 (Agilent Technologies). The Agilent SurePrint G3 Human Gene Expression version 3 8 × 60K Microarrays were used in this experiment. The sample labeling, microarray hybridization and washing were performed based on the manufacturer‘s standard protocols. Briefly, total RNAs were transcribed to double-stranded cDNA, then synthesized into cRNA and labeled with Cyanine-3-CTP. The labeled cRNAs were hybridized onto the microarray. After washing, the arrays were scanned by the Agilent Scanner G2505C (Agilent Technologies).

Feature Extraction software (version 10.7.1.1, Agilent Technologies) was used to analyze array images to get raw data. Next, the raw data was normalized with the quantile algorithm. The probes detected with 3 samples in any group were chosen for further data analysis. Differentially expressed genes were then identified through fold change as well as *P* value calculated with *t* test. The threshold set for up- and downregulated genes was log2 fold change greater than 0.5 and *P* value less than 0.05. Afterwards, KEGG analysis was applied to determine the roles of these differentially expressed mRNAs. The microarray data have been stored in the Gene Expression Omnibus (GEO). Data can be accessed through http://www.ncbi.nlm.nih.gov/geo (accession number: GSE183786).

### Statistics.

The Statistical Package for the Social Sciences version 17.0 software and Prism 8 (Graphpad) software were used for statistical analysis. Comparison between 2 experimental groups was determined with 2-tailed Student’s *t* test. More than 2 groups were made using ANOVA followed by Tukey’s honestly significant difference (HSD) test. Data in this study represent at least 3 independent experiments and are presented as mean ± SEM. Results were considered significant at *P < 0.05 and **P < 0.01, and specific comparisons are indicated in the respective figure legends.

### Study approval.

All experiments were conducted in accordance with the Association for Research in Vision and Ophthalmology Statement for the Use of Animals in Ophthalmic and Vision Research, and they were approved by the Ethics Committee of Shandong Eye Institute (accreditation no. 2012-G-D01).

## Author contributions

WS and QZ conceived and designed the project, interpreted results, and wrote the manuscript. ZL performed experiments, analyzed data, and wrote the manuscript. HD performed cell experiments and immunostaining experiments. WL and BM performed cell experiments. YJ, CZ, XW, YG, and CD performed animal experiments. SD and BZ analyzed data. DL performed animal examination. LX and YC revised the manuscript. ZL is listed first in the order of co–first authors because of his major contributions to research design and manuscript preparation.

## Supplementary Material

Supplemental data

## Figures and Tables

**Table 2:**
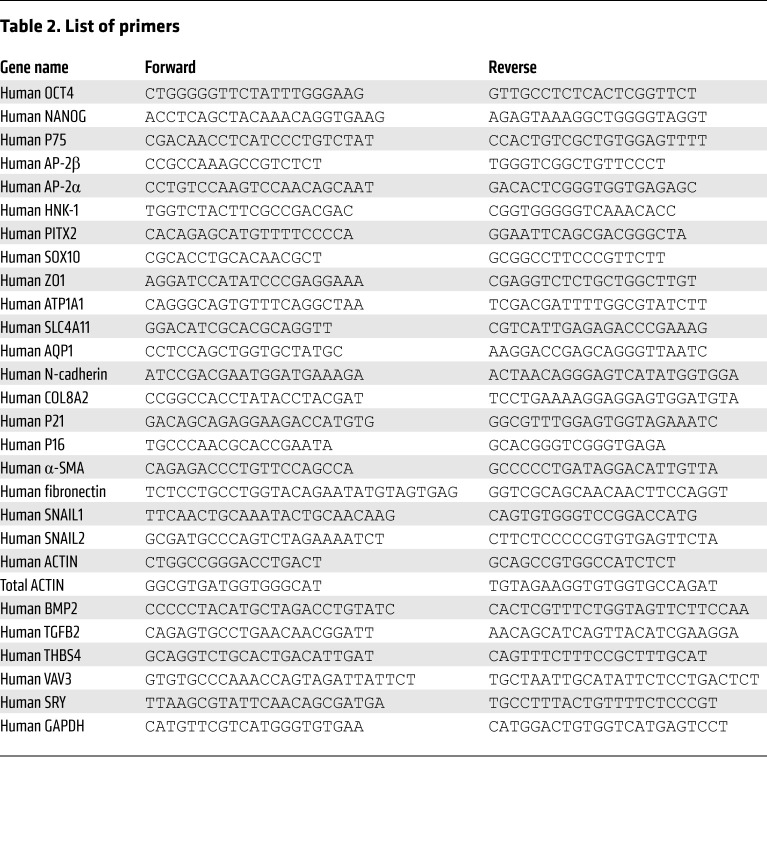
List of primers

**Table 1:**
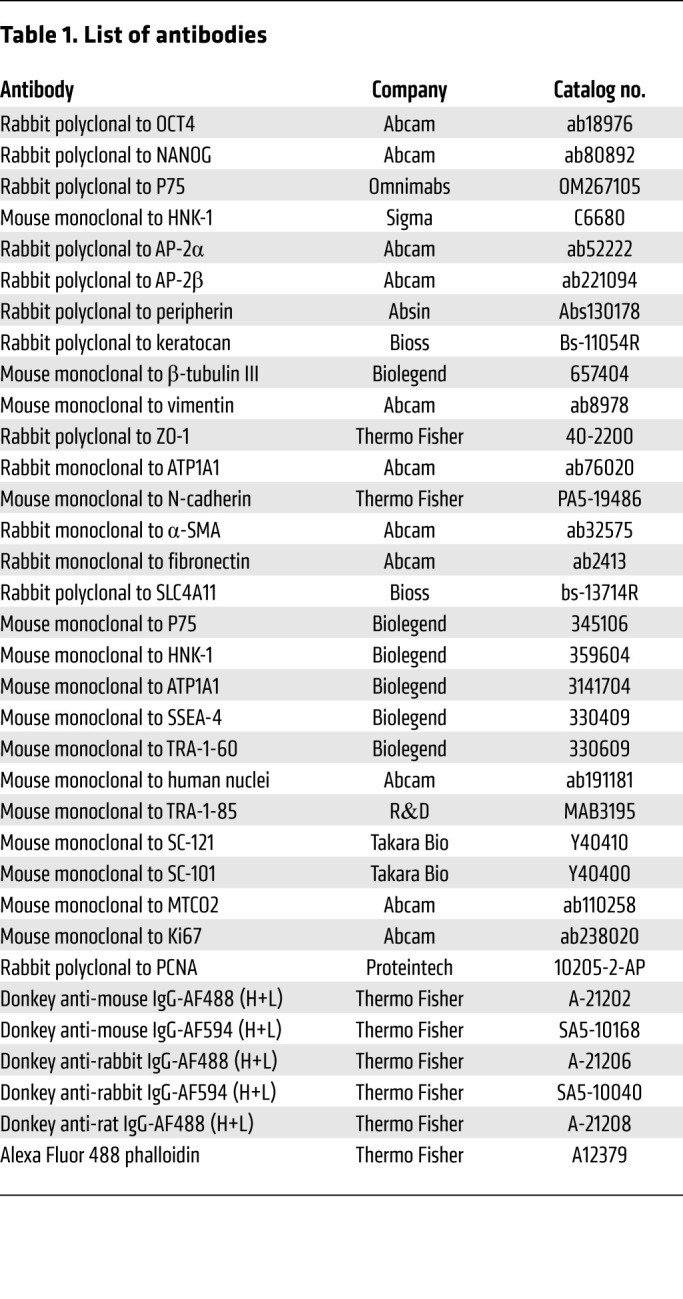
List of antibodies

## Version 1 01/04/2022

Electronic publication
