# Supplementary material for: Long-term corneal recovery by simultaneous delivery of hPSC-derived corneal endothelial precursors and nicotinamide
Source: J Clin Invest. 2022 Jan 4;132(1):e146658. doi: 10.1172/JCI146658 (PMC8718141; doi:10.1172/JCI146658)
Supplement: Supplemental data [file jci-132-146658-s130.pdf]

Supporting Information

Supplemental Figure 1

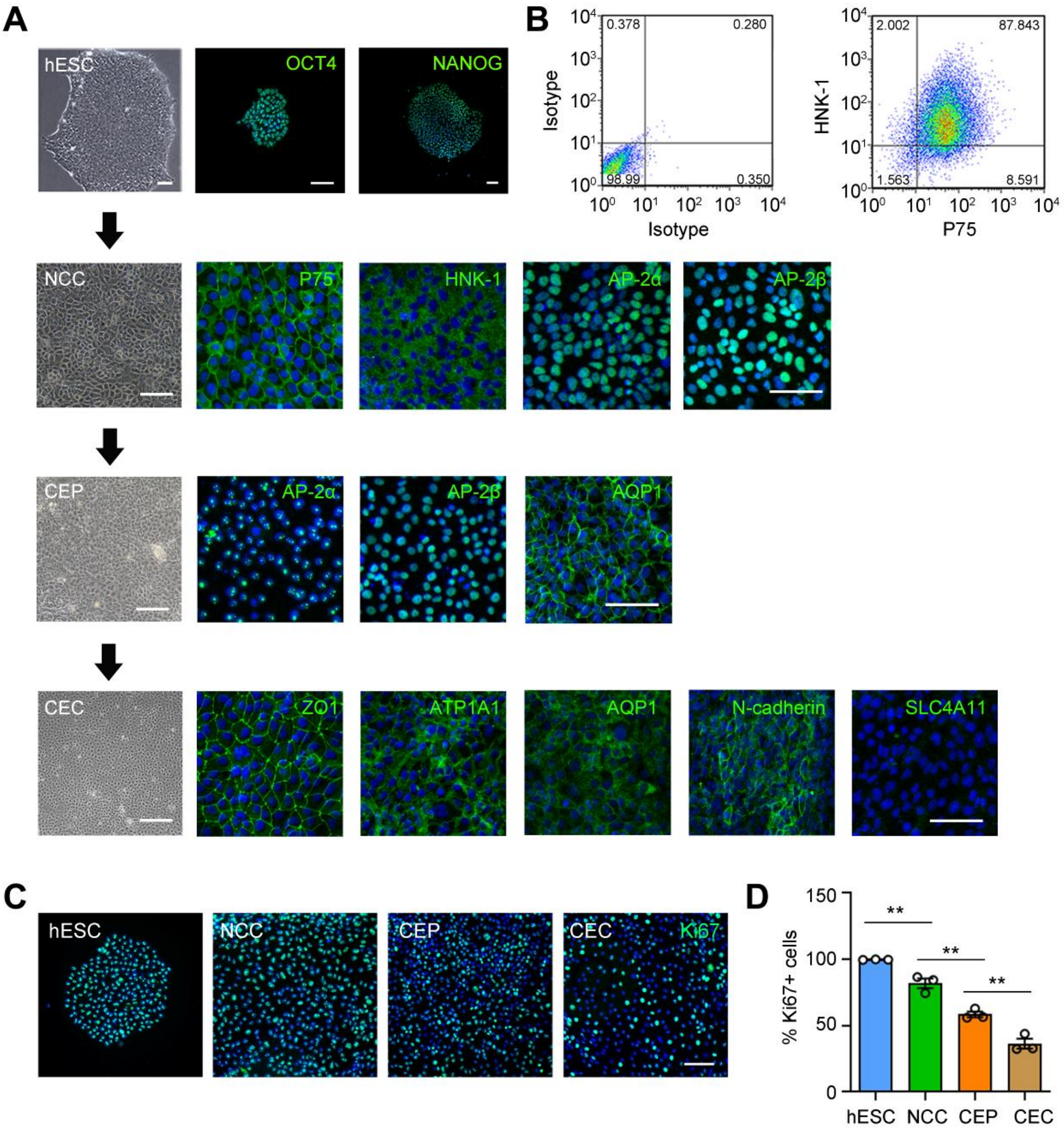

Supplemental Figure 1. Characterizations of hESCs and differentiated cells. (**A**) Representative morphology and immunostaining of hESCs, NCCs, CEPs and CECs. OCT4 and NANOG as the markers of hESCs, P75 and HNK-1 as the markers of NCCs, AP-2α and AP-2β as the markers of NCCs and CEPs, ZO1, ATP1A1, AQP1, N-cadherin and SLC4A11 as the markers of CECs. Nuclei were stained with DAPI. Scale bar: 100 μm (morphology); Scale bar: 50 μm (immunostaining). (**B**) Flow cytometry analysis of hESC-derived NCCs. The experiments were repeated three times. (**C**) Ki67 immunostaining of hESCs and differentiated cells. Nuclei were stained with DAPI. Scale bar: 50 μm (**D**) Statistical analysis of the percentage of Ki67 positive cells. n = 3, $^{**}P < 0.01$ by one-way ANOVA with Tukey's HSD test.

Supplemental Figure 2

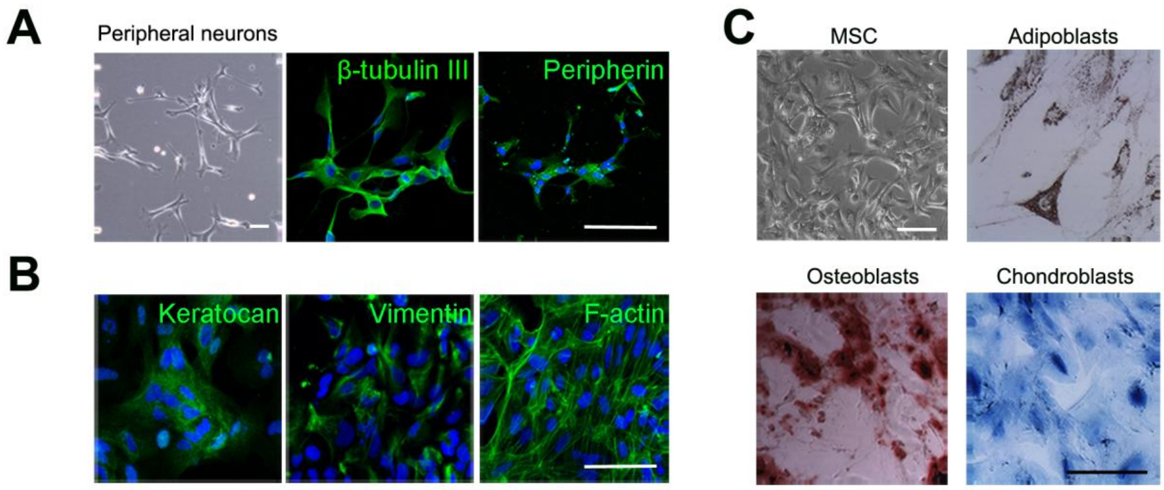

Supplemental Figure 2. Multipotent capacity of hESC-derived NCCs. (**A**) Morphology and immunostaining of induced peripheral neuron from hESC-derived NCCs. Scale bar: 50 μm. (**B**) Immunostaining of induced corneal keratocyte from hESC-derived NCCs. Nuclei were stained with DAPI. Scale bar: 50 μm. (**C**) Morphology and multipotent differentiation of induced mesenchymal stem cells (MSCs) from hESC-derived NCCs. The differentiated adipoblasts, osteoblasts and chondroblasts were identified with Oil Red O, Alizarin Red S and Alcian Blue staining. Scale bar: 100 μm.

Supplemental Figure 3

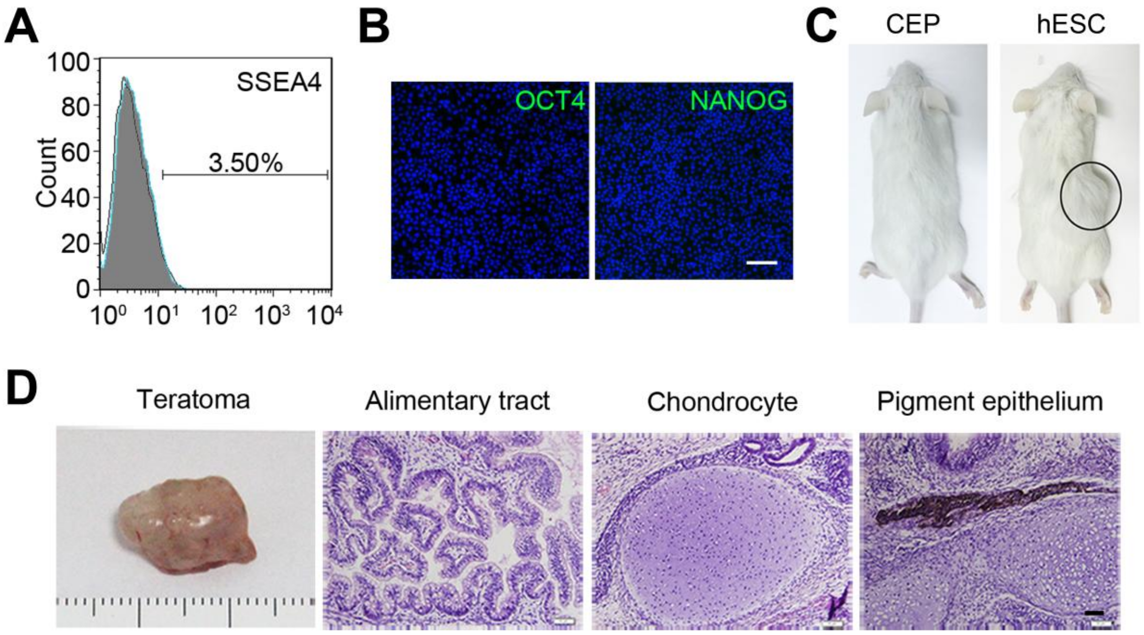

Supplemental Figure 3. Safety of hESC-derived CEPs. (**A**) Flow cytometry analysis of SSEA4 staining. The experiments were repeated three times. (**B**) OCT4 and NANOG immunostaining of hESC-derived CEPs. Nuclei were stained with DAPI. Scale bar: 50 µm. (**C**) Tumorigenesis test of hESCs and hESC-derived CEPs. No teratomas in 10 mice after 3 months of hESC-derived CEPs injection, while 9 of 10 mice with tumor formation after hESC injection as shown in black circle. (**D**) The teratomas of mice with hESC injection were isolated and identified with hematoxylin-eosin staining. The three germ layers, including endoderm-derived alimentary tract, mesoderm-derived chondrocyte and ectoderm-derived pigment epithelium were found. Scale bar: 50 µm.

Supplemental Figure 4

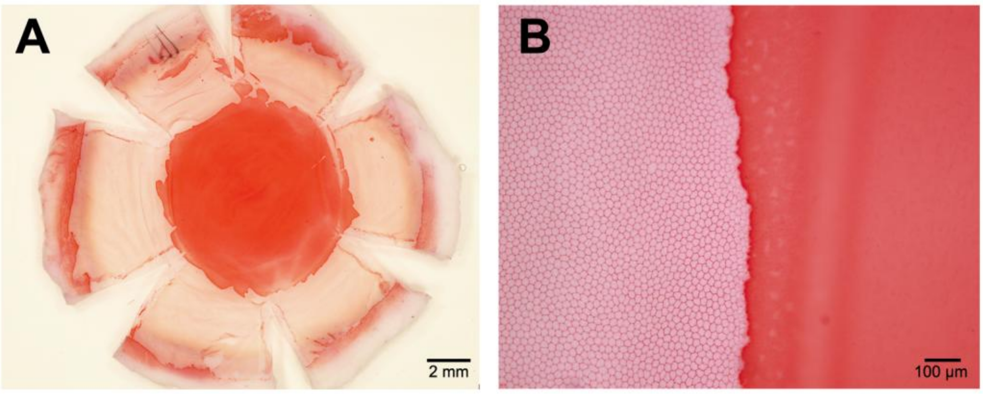

Supplemental Figure 4. Representative staining of rabbit scraped corneal endothelium. (**A**) Whole-mounted corneal Alizarin red staining. Scale bar: 2 mm; (**B**) Magnified image of adjacent area between scraped area (red) and resident area. Scale bar: 100 μm.

Supplemental Figure 5

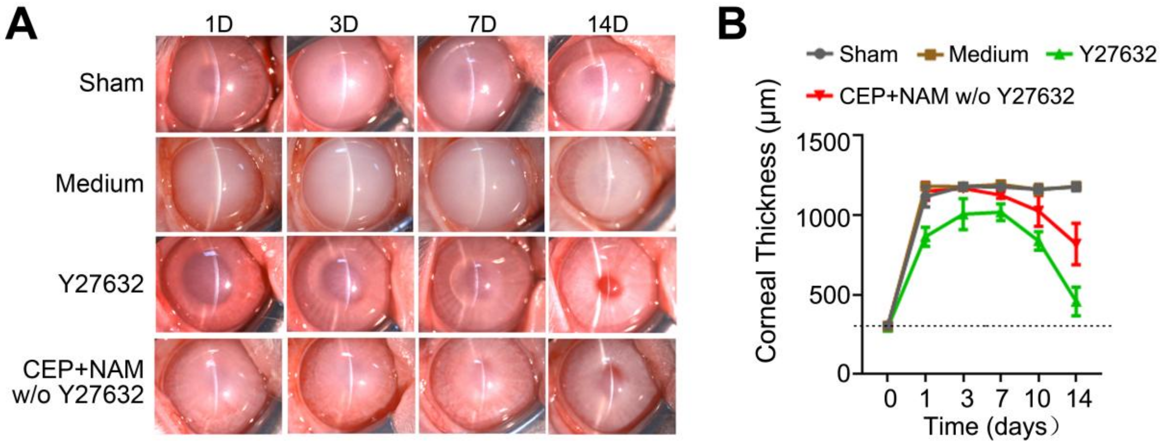

Supplemental Figure 5. Representative images of rabbit corneas after operation. The rabbits were performed with sham operation or intracameral injection of simple medium, Y27632 alone, CEP and NAM without Y27632 treatment (CEP+NAM w/o Y27632). The changes of corneal clarity were observed by slit-lamp microscopy. (**B**) Corneal thicknesses were measured by pachymeter 1, 3, 7, 10 and 14 days after transplantation. n = 3. The dashed line shows normal corneal thickness.

Supplemental Figure 6

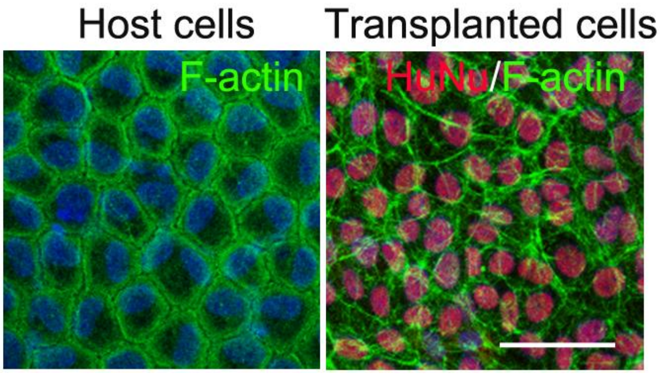

Supplemental Figure 6. F-actin and HuNu immunostaining of corneal endothelium in rabbits with CEP and NAM treatment 7 days later. Nuclei were stained with DAPI. Scale bar: 50 μm.

Supplemental Figure 7

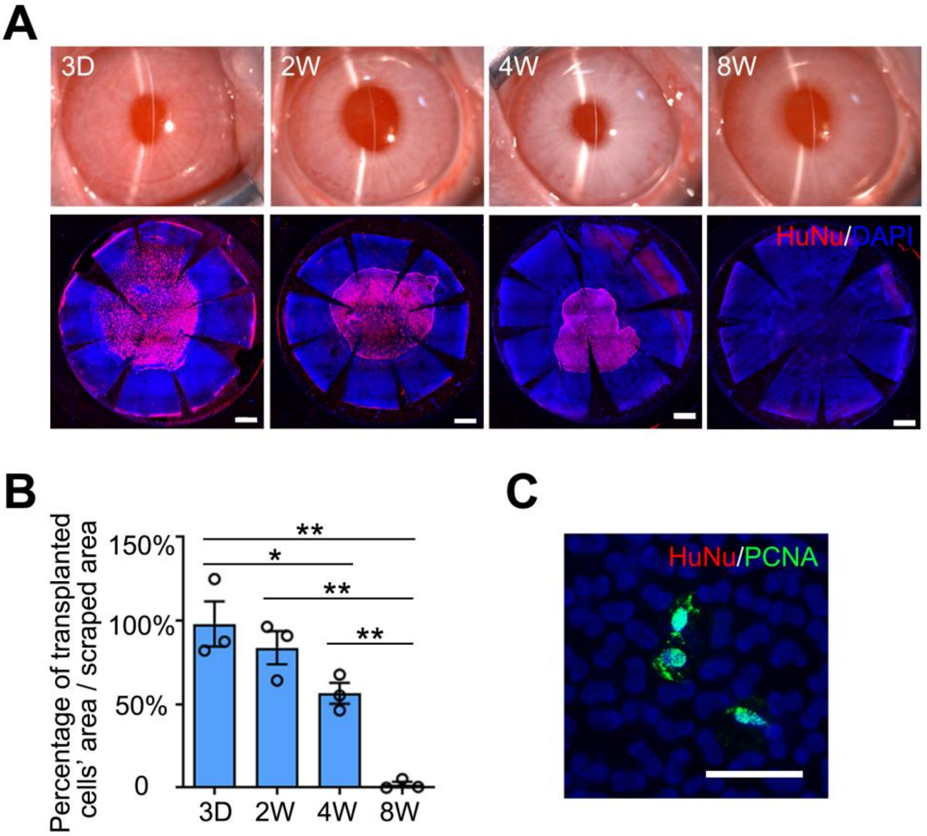

Supplemental Figure 7. Analysis of transplanted cell survival and host cell regeneration in rabbits with CEP transplantation and NAM treatment. (**A**) Slit-lamp microscopy images and HuNu immunostaining after 3 days and 2-8 weeks of transplantation. Nuclei were stained with DAPI. Scale bar: 2000 μm. (**B**) Statistical analysis of transplanted CEP-covered endothelial area. The 9-mm diameter scraped area was calculated as 100%. n = 3, $^{**}P < 0.01$, $^{*}P < 0.05$ by one-way ANOVA with Tukey's HSD test. (**C**) PCNA immunostaining of rabbit peripheral corneal endothelium after 14 days of CEP and NAM treatment. Nuclei were stained with DAPI. Scale bar: 50 μm

Supplemental Figure 8

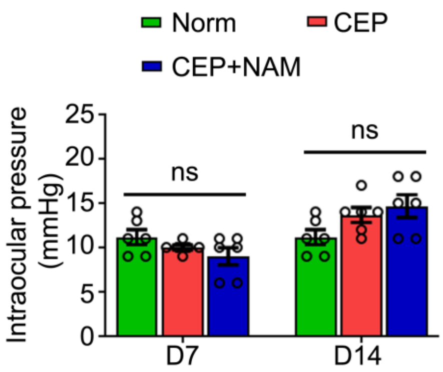

Supplemental Figure 8. Intraocular pressure measurement of rabbit models after 7 and 14 days of CEP transplantation with or without NAM treatment, normal rabbits as control. n=6. one-way ANOVA with Tukey's HSD test. ns means no significance.

Supplemental Figure 9

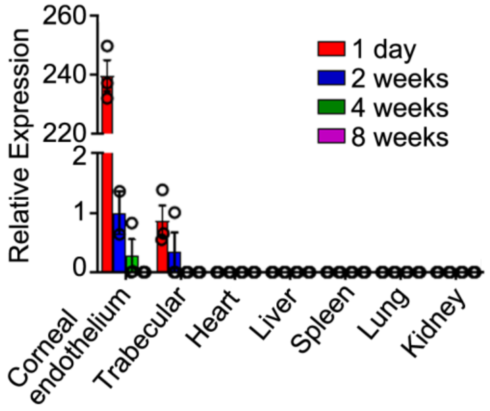

Supplemental Figure 9. Expressions of human specific *Actin* in corneal endothelium, trabecular, heart, liver, spleen, lung and kidney were analysed after 1 day and 2-8 weeks of CEP transplantation and NAM treatment. Normalized to total *Actin* expression of human and rabbit, n = 3.

Supplemental Figure 10

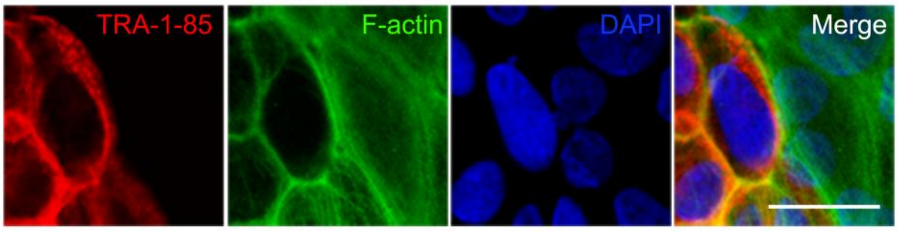

Supplement Figure 10. Integration of host and transplanted cells in rabbits. F-actin (green) and human cell surface determinant TRA-1-85 (red) staining of rabbit corneal endothelium after 14 days of CEP transplantation and NAM treatment. Nuclei were stained with DAPI. Scale bar: 20 μm.

Supplemental Figure 11

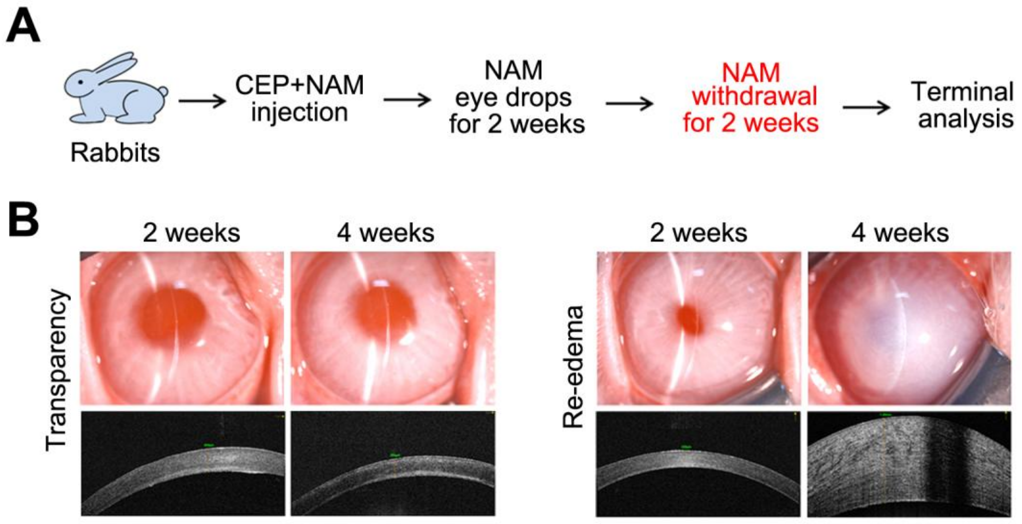

Supplemental Figure 11. Effects of incontinuous NAM treatment for corneal recovery in rabbit. (**A**) Experimental design of incontinuous NAM treatment. (**B**) Representative images of slit-lamp microscopy and anterior segment OCT of rabbits before and after NAM withdrawal. Six of the ten rabbits persisted the transparent cornea, and four became re-edema.

Supplemental Figure 12

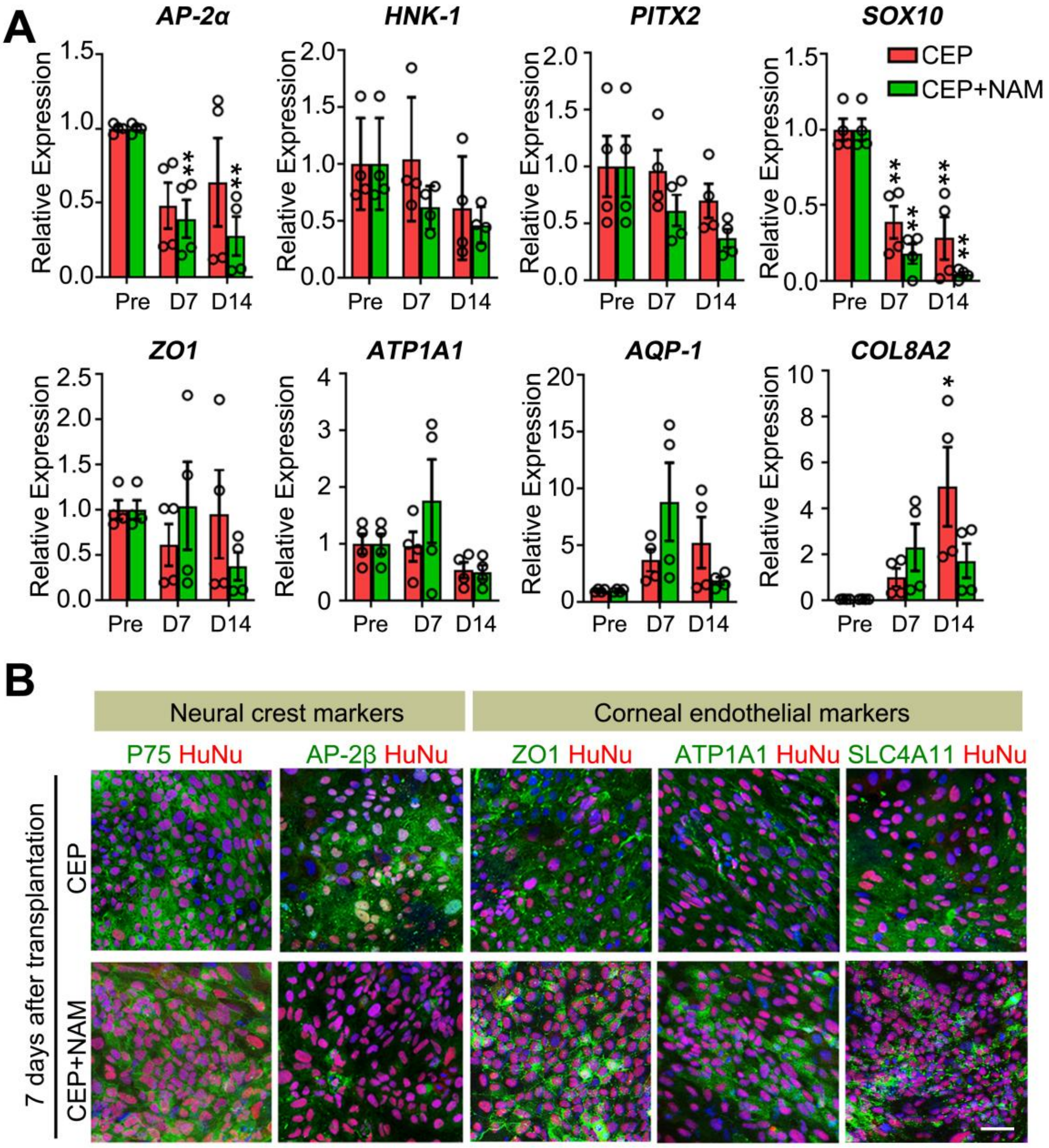

Supplemental Figure 12. Characterization of CEPs before and after transplantation. (**A**) qPCR analysis of neural crest and corneal endothelial gene expression in the pretransplanted (Pre) and CEPs after 7 and 14 days of transplantation. n = 4. **P* <0.05, ***P* <0.01 versus pretransplanted CEPs. One-way ANOVA with Tukey's HSD test. (**B**) P75, AP-2β, ZO-1, ATP1A1 and SLC4A11 immunostaining of rabbit corneal endothelium after 7 days of CEP transplantation with or without NAM treatment. Transplanted human cells were stained with HuNu. Nuclei were stained with DAPI. Scale bar: 50 μm.

Supplemental Figure 13

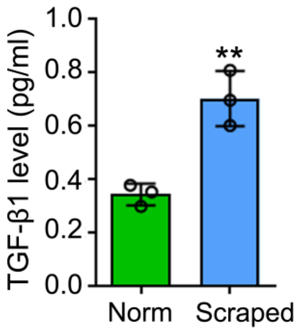

Supplemental Figure 13. TGF-β1 measurement of aqueous humor in normal and endothelium-scraped rabbits. n = 3, ***P* <0.01 by 2-tailed Student's t test.

Supplemental Figure 14

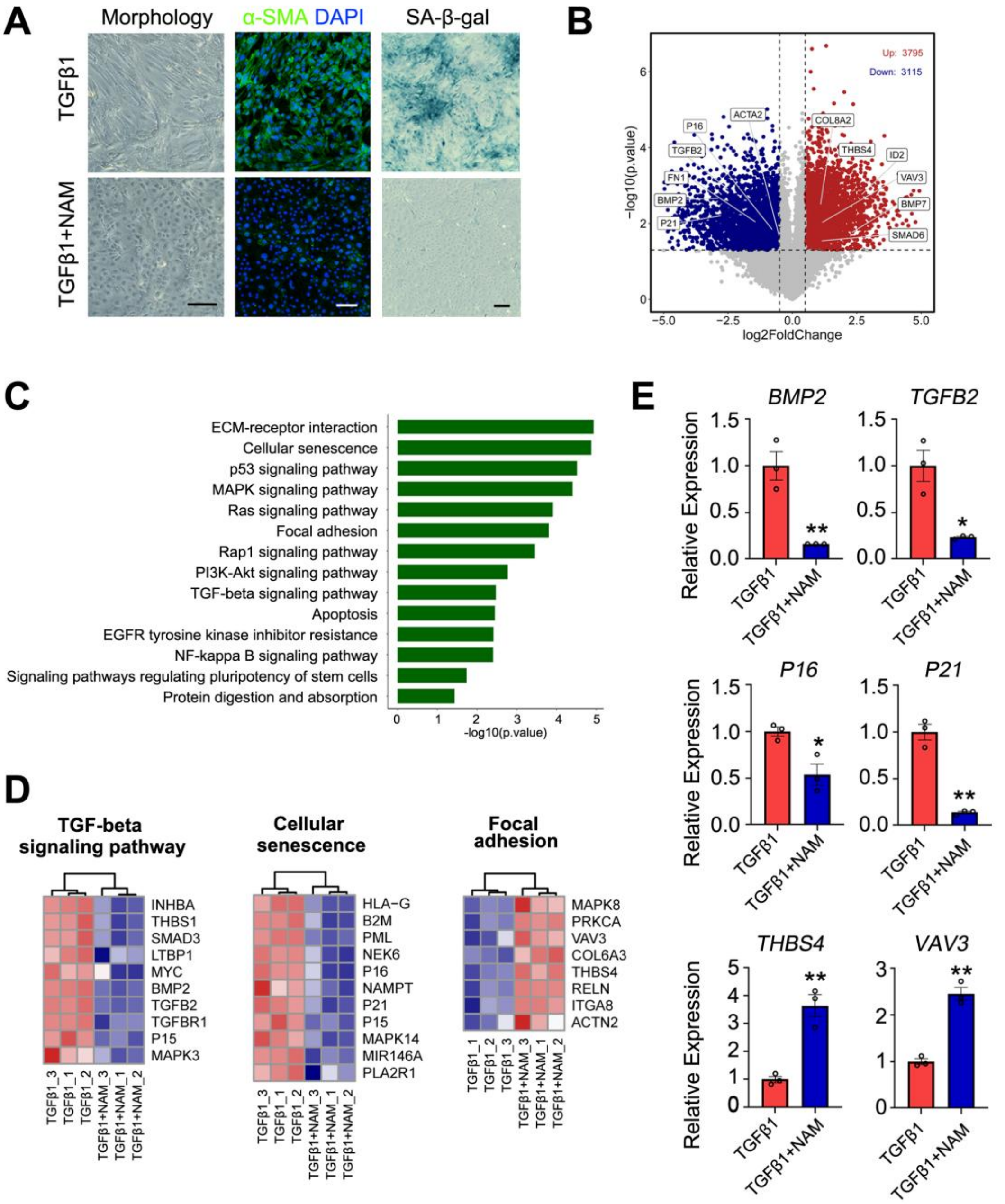

Supplement Figure 14. Regulation of NAM on EnMT, senescence and gene expression of TGFβ1-treated CEPs. (**A**) Induced CEPs were treated with 30 ng/ml TGFβ1 with or without 5 mM NAM for 3-5 days in vitro. The morphology changes, α-SMA immunostaining and SA-β-gal staining were compared. Scale bar: 100 μm. (**B**) Volcano plot of dysregulated and representative genes. (**C**) Representative KEGG terms for differentially expressed genes. (**D**) Representative gene clusters changed with NAM treatment. (**E**) qPCR validation of representative genes. n = 3. *P <0.05, **P <0.01 by 2-tailed Student's t test.

Supplemental Figure 15

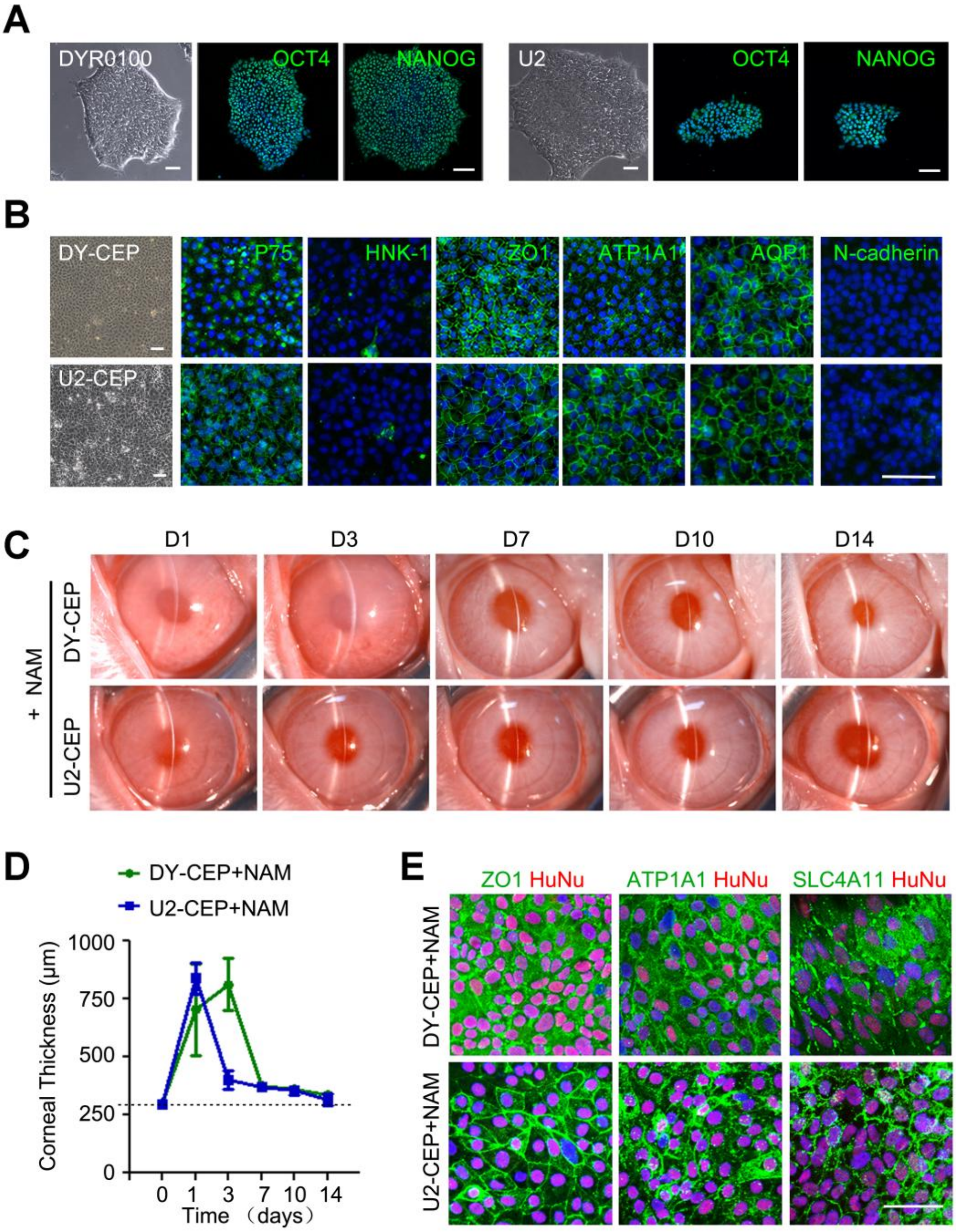

Supplemental Figure 15. Efficacy of hiPSC-derived CEPs with NAM treatment for corneal recovery in rabbits. (**A**) Morphology and identification of hiPSC cell line DYR0100 and U2. Nuclei were stained with DAPI. Scale bar: 100 μm. (**B**) Morphology and identification of hiPSC-derived CEPs. Nuclei were stained with DAPI. Scale bar: 50 μm. (**C**) Representative images of rabbit corneas after 1-14 days of hiPSC-derived CEP transplantation and NAM treatment. (**D**) Corneal thicknesses measurement. n = 3. The dashed line shows normal corneal thickness. (**E**) ZO1, ATP1A1 and SLC4A11 immunostaining of rabbit corneal endothelium after 14 days of transplantation. Transplanted cells were stained with HuNu. Nuclei were stained with DAPI. Scale bar: 50 μm.

Supplemental Figure 16

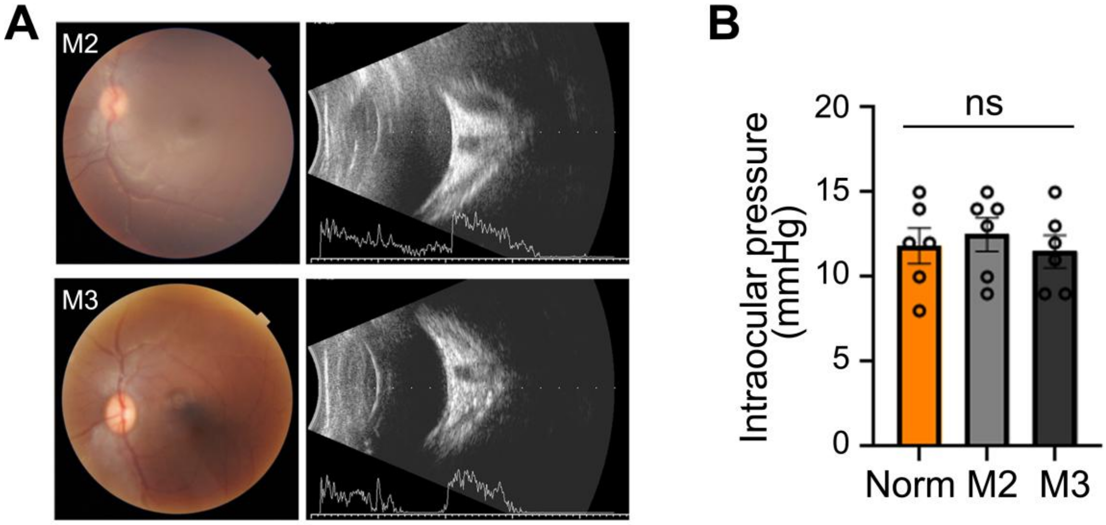

Supplement Figure 16. (**A**) Fundus photography and B-mode ultrasound examinations of the monkeys (M2 and M3) after 3 months of CEP transplantation and NAM treatment. (**B**) Intraocular pressure measurement. one-way ANOVA with Tukey's HSD test. ns means no significance.

Supplemental Figure 17

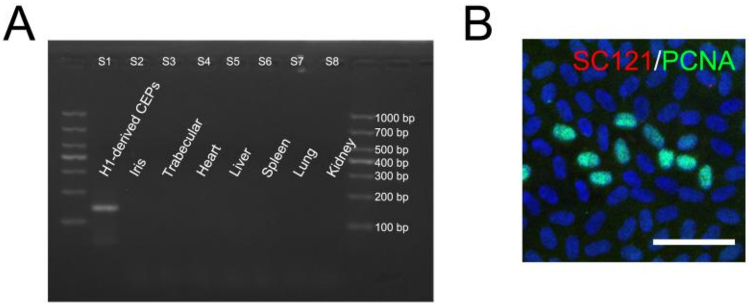

Supplemental Figure 17. (A) PCR of the male sex-determining gene SRY expression in female monkey tissues after 36 months of CEP transplantation and NAM treatment. H1-derived male CEPs as positive control. (**B**) PCNA immunostaining of monkey resident corneal endothelium after 3 months of CEP transplantation and NAM treatment. Nuclei were stained with DAPI. Scale bar: 50 μm.

Supplemental Figure 18

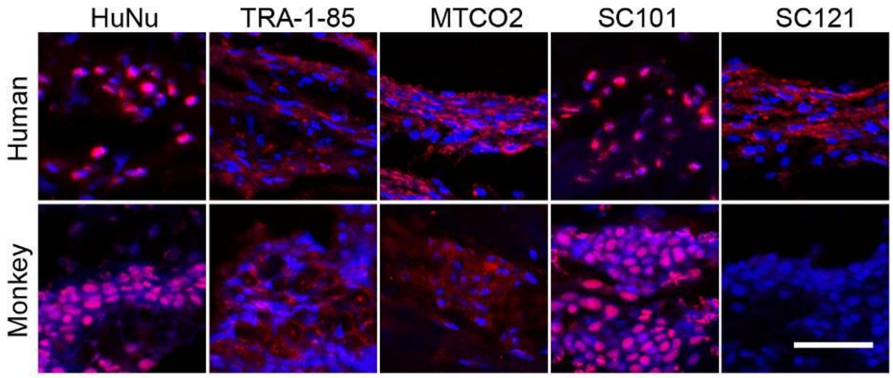

Supplemental Figure 18. Antibody selection for discriminating human from non-human primate cells. Human and monkey conjunctival tissues were stained with HuNu, TRA-1-85, MTCO2, SC101 and SC121. Nuclei were stained with DAPI. Scale bar: 50 μm
